# In silico identification of compounds from *Nigella sativa* seed oil as potential inhibitors of SARS-CoV-2 targets

**DOI:** 10.1186/s42269-021-00517-x

**Published:** 2021-03-12

**Authors:** Chidi Edbert Duru, Ijeoma Akunna Duru, Abayomi Emmanuel Adegboyega

**Affiliations:** 1 Surface Chemistry and Environmental Technology (SCENT) Research Unit, Department of Chemistry, Imo State University, Owerri, Imo State Nigeria; 2 Department of Chemistry, Federal University of Technology, Owerri, Imo State Nigeria; 3 Department of Biochemistry, Faculty of Medical Sciences, University of Jos, Jos, Plateau State Nigeria; 4 Africa Center of Excellence in Phytomedicine Research and Development, University of Jos, Jos, Plateau State Nigeria

**Keywords:** *Nigella sativa*, Angiotensin-converting enzyme, SARS-CoV-2, β-bisabolene, Binding affinity

## Abstract

**Background:**

The growing number of cases, severity and fatality of the COVID-19 pandemic, coupled with the fact that no cure has been found has made infected individuals especially in Africa, to resort to the consumption of different natural products to alleviate their condition. One of such plant materials that have been consumed to remedy the severity of this viral infection is the oil of *Nigella sativa* seed commonly called black seed oil. In this study, we extracted and characterized the oil from this seed using gas chromatography coupled to a mass selective detector to identify the component phytochemicals. Site-directed multiligand docking of the identified compounds was performed on SARS-CoV-2 molecular targets- Replicase polyprotein 1a, RNA binding protein of NSP9, ADP ribose phosphatase of NSP3, 3-chymotrypsin-like protease 3CLpro, and RNA-dependent RNA polymerase RDRP, and ACE2–angiotensin-converting enzyme from the Homo sapiens.

**Results:**

The binding affinity of caryophyllene oxide was the highest on 3CLpro (− 6.0 kcal/mol), NSP3 (− 6.3 kcal/mol), NSP9 (− 6.3 kcal/mol), and RDRP (− 6.9 kcal/mol) targets, while α-bergamotene gave the best binding affinity on RPIA (5.7 kcal/mol) target. The binding affinity of β-bisabolene on the ACE2 target (− 8.0 kcal/mol) was almost the same as Remdesivir (− 8.1 kcal/mol). The ADMET properties of these three phytochemicals showed that they are good drug leads for these SARS-CoV-2 receptors.

**Conclusion:**

The findings from this study strongly indicate that the reported recovery from COVID-19 infection claimed by patients who consumed black seed oil could be linked to the presence of caryophyllene oxide, α-bergamotene, and β-bisabolene in this natural product.

## Background

Black cumin, scientifically known as *Nigella sativa* L., is an annual dicotyledonous herb that belongs to the family Ranunculaceae (Datta et al. 2012). It is a small shrub ranging between 20 and 90 cm in height, with tapering green leaves and rosaceous pink, purple, white, yellow, or pale blue flowers with 5–10 petals. Flowering starts in July and the seeds ripen in September. The ripe fruit is a capsule with 3–7 united follicles containing numerous tiny, pitch-black seeds with an aromatic and slightly bitter taste (Ahmad et al. 2013). It is native to northeastern Africa, the eastern Mediterranean, and southwestern Asia (Vavilov and Dorofeev 1992). The species is grown in many countries like India, Turkey, Middle East, Pakistan, Syria, and Saudi Arabia (Naz 2011). The seeds of *N. sativa* have been widely used as diuretics, anti-diarrheal, analgesic, antibacterial, antidiabetic, anticancer, immunomodulator, anti-inflammatory, spasmolytic, bronchodilator, hepatoprotective, renal protective, gastro-protective, antioxidant, etc. (Ahmed et al. 2013; Sultan et al. 2014).

The chemical composition of seed extracts of *N. sativa* seed varies from one country to another and also from one region in a country to another (Shariq et al. 2015). The major components in *N. sativa* seed are fatty oils and aliphatic hydrocarbons, arachidonic acid, tocopherols, γ-linolenic acid, and essential oil, which contains carvacrol, ρ-cymene, thymoquinone, α-pinene, and indazole-type alkaloids, isoquinoline alkaloids, as well as dolabellane-type diterpene alkaloids. The food and therapeutic applications of *N. sativa* oil and seed have a long history in many cultures. Islamic medicine also cataloged the numerous benefits of this plant (Sharma et al. 2009). Its compounds have been shown to have various pharmacological effects on different body parts. Empirical studies have reported that the seed oil and seed extracts have antimicrobial activities, which some workers have attributed to the presence of compounds such as thymohydroquinone and melanin (Kalim 2013; Al Yahya 1986). The seed extract contains numerous antioxidants like carvacrol, t-anethole, and 4-terpineol, and their activities have been reported in different studies (Awadalla 2012). Also, available reports have shown the anti-inflammatory, anticancer, anti-diabetic, and hepatoprotective activities of its oil and extracts (Umar et al. 2012; Peng et al. 2013; Abdelmeguid et al. 2010).

The Coronavirus disease 2019 (COVID-19) caused by the severe acute respiratory syndrome Coronavirus 2 (SARS-CoV-2) began in Wuhan, China, and was reported to the World Health Organization on December 31, 2019 (WHO 2020). The pandemic ravaged the entire globe, claiming hundreds of thousands of lives. Community to community transmission is now evident in many countries, with a daily rise in the number of cases. There are potential drug targets essential for the survival of the coronavirus, which include a 3-chymotrypsin-like protease (3CLpro), Angiotensin-converting enzyme (ACE2), ADP ribose phosphatase of Nonstructured Protein 3 (NSP3), Nonstructured Protein 9 RNA binding protein (NSP9), RNA-dependent RNA polymerase (RdRp), and Replicase Polyprotein 1a (RP1a). To infect the host, the structural spike glycoprotein interacts with the transmembrane protein of the human host cell receptor ACE2 (Guy et al. 2005). The main protease 3CLpro acts in the viral polyprotein large Replicase Polyprotein 1a (RP1a) proteolytic cleavage, cleaving it into functional units to produce nonstructural viral constituents like RdRp, NSP3, and NSP9. This makes 3CLpro and ACE2 important drug targets to inhibit Nonstructured Proteins production and terminate viral action. The RP1a and the NSPs (RdRp, NSP3, and NSP9) are important targets involved in the replication and translation process, which lead to a viral proliferation in host cells (Kumar et al. 2020). In vitro and in silico studies of possible cures for this dreaded disease have become hot topics in current research. Although no drug is currently available to cure this disease, Remdesivir, an antiviral drug has been clinically tested and confirmed useable in the interim (Williamson et al. 2020).

Recently, the governor of Oyo State in Nigeria revealed that the oil from *N. sativa* seed was one of the remedies he consumed to combat the disease, a week after he tested positive for COVID-19 (Punch Newspaper, April 7, 2020). So far, no attempt has been made to investigate the veracity of this claim. In this study, the oil from the seed of *N. sativa* was extracted, and the identified phytochemical components docked on some selected SARS-CoV-2 proteins. The dock scores of these compounds were compared to the values obtained with a standard COVID-19 prescription drug.

## Methods

### Sample collection and preparation

Mature seeds of *N. sativa* were collected toward the end of rainy season in October 2020 (average temperature 29 °C), from a fallow farmland in Northern Nigeria (N 10° 9′ 32.2524", E 8° 8′ 1.8816"), and no permissions or licenses were required for the collection and study. They were identified by a professional taxonomist Prof. F.N. Mbagwu of the Department of Plant Science and Biotechnology, Imo State University, Owerri, Nigeria, and the voucher specimen was deposited at the Imo State University Herbarium, with herbarium number IMSUH- 468. They were washed with tap water and left to dry on a laminated paper for one week under room conditions. The dried seeds were subjected to preliminary grinding using mortar and pestle, and the particle size was further reduced using an electric blender. The seed powder was transferred in an airtight plastic container and stored in a refrigerator at 4 °C.

### Extraction of oil

Oil was extracted from the ground seed powder using a Soxhlet apparatus mounted on a heating mantle (Duru 2020). The seed powder of weight 100 g was encapsulated in a clean white cotton cloth previously degreased in hexane (≥ 98.5%, Sigma-Aldrich, Darmstadt, Germany). The sample was held in the thimble-holder of the extractor which was gradually filled with condensed redistilled hexane from a distillation flask placed on a heating mantle. When the solvent reaches the overflow level in the thimble-holder, it is siphoned back into the distillation flask, carrying the extracted oil with it. The operation was repeated until complete extraction was achieved. The extracted oil was then recovered from the solvent by rotary evaporation.

### Characterization of oil

The oil sample was characterized using gas chromatography-mass Spectrometer (GC–MS) instrument (Model: 7890 GC and 5977B MSD, Agilent Technologies, USA). An HP-5 MS capillary standard nonpolar column L × I.D., 30 m × 0.25 mm and film thickness 0.25 µm was used. The flow rate of mobile phase (carrier gas: He) was set at 1.0 mL/min. In the gas chromatography part, the temperature program (oven temperature) was set at 40 °C and raised to 250 °C at 5 °C/min and injection volume 1 µL. The oil sample was dissolved in methanol (≥ 99.8%, Sigma-Aldrich, Darmstadt, Germany) and fully scanned at a range of 40–650 m/z, and the results were compared using NIST mass spectral library search program (El-Sawi et al. 2020).

### Identification and preparation of ligands

The 3D structure-data files (SDF) of the compounds in the crude oil sample were identified and downloaded from the PubChem database. They were minimized in PyRx virtual screening tool, using Universal Force Field at 200 steps. They were then converted to AutoDock ligands (pdbqt) and used for the docking analysis.

### Identification and preparation of molecular targets

**Fig. 1:**
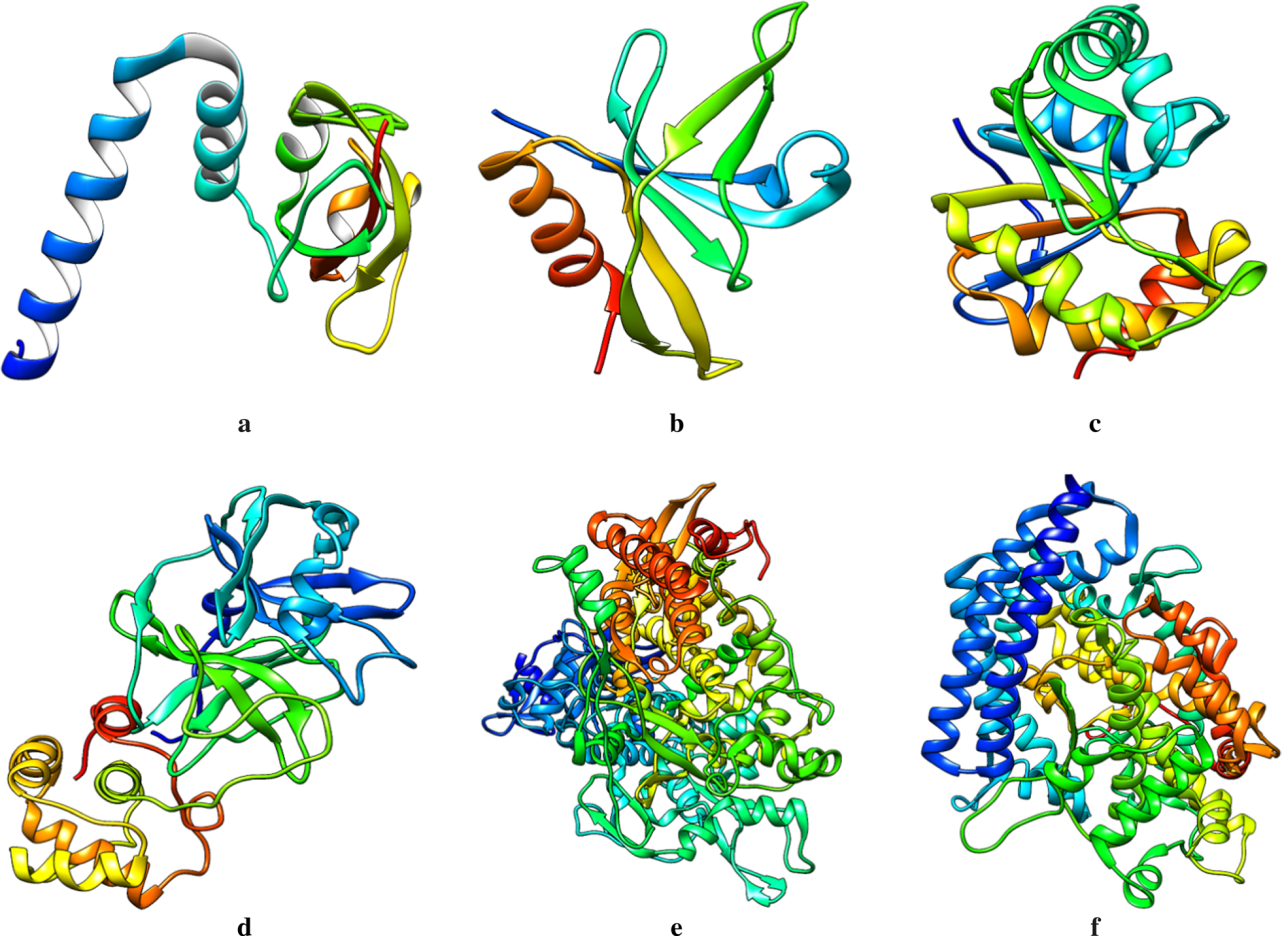
Crystal structure of prepared molecular targets **a** Replicase polyprotein 1a. **b** RNA binding protein of NSP9. **c** ADP ribose phosphatase of NSP3. **d** 3-chymotrypsin-like protease. **e** RNA-dependent RNA polymerase. **f** Angiotensin-converting enzyme

Five SARS-CoV-2 molecular targets (Fig. 1), Replicase polyprotein 1a (6YHU), RNA binding protein of NSP9 (6W4B), ADP ribose phosphatase of NSP3 (6VXS), 3-chymotrypsin-like protease 3CLpro (6LU7) and RNA-dependent RNA polymerase RDRP (7BTF), and one protein of host, ACE2–Angiotensin-converting enzyme (6LZG), were identified from literature (Konkolova et al. 2020; Tan et al. 2020; Michalska et al. 2020; Jin et al. 2020; Gao et al. 2020; Wang et al. 2020) and downloaded from the Protein Data Bank (PDB). The interfering crystallographic water molecules and cocrystallized ligand were removed, and minimization of the energy of the protein was then done using UCSF Chimera 1.14 (Pettersen 2004; Duru et al. 2020). The protein was minimized at 300 steepest descent steps at 0.02 Å. The conjugate gradient steps were 10 at 0.02 Å and 10 update intervals. Gasteiger charges were added using Dock Prep to get a good structure conformation.

### Determination of active sites on SARS-CoV-2 proteins

The active sites of the proteins were determined using CASTp (Computed Atlas for Surface Topography of Proteins).

(Tian et al. 2018; http://sts.bioe.uic.edu/castp/index.html?j_5f45dd381f58d).

### Docking procedure and analysis of results

The screening of the phytochemical compounds from the oil was performed by docking them on selected binding pockets of SARS-CoV-2 proteins and ranked based on their binding energies. The multiple docking of the ligands and proteins was done with Autodock Vina in PyRx software (Tsao et al. 2020; Johnson et al. 2020). The molecular docking results were organized on an Excel spreadsheet, and the Heat Map of the data was viewed using the Conditional Formatting function.

### Absorption, distribution, metabolism and elimination and toxicity (ADMET) analysis

The compounds with the lowest binding energy for each protein were selected and submitted to the ADMETsar 2 server to examine their drug-like properties, pharmacokinetics, and pharmacodynamics parameters (Yang et al. 2019).

### Analysis of protein–ligand interactions

The interactions between the most potent compounds in the oil and amino acid residues of the proteins were visualized using Biovia Discovery studio client 20.1 (BIOVIA 2020) and UCSF Chimera software.

## Results

### Chemical composition of oil extract

**Fig. 2:**
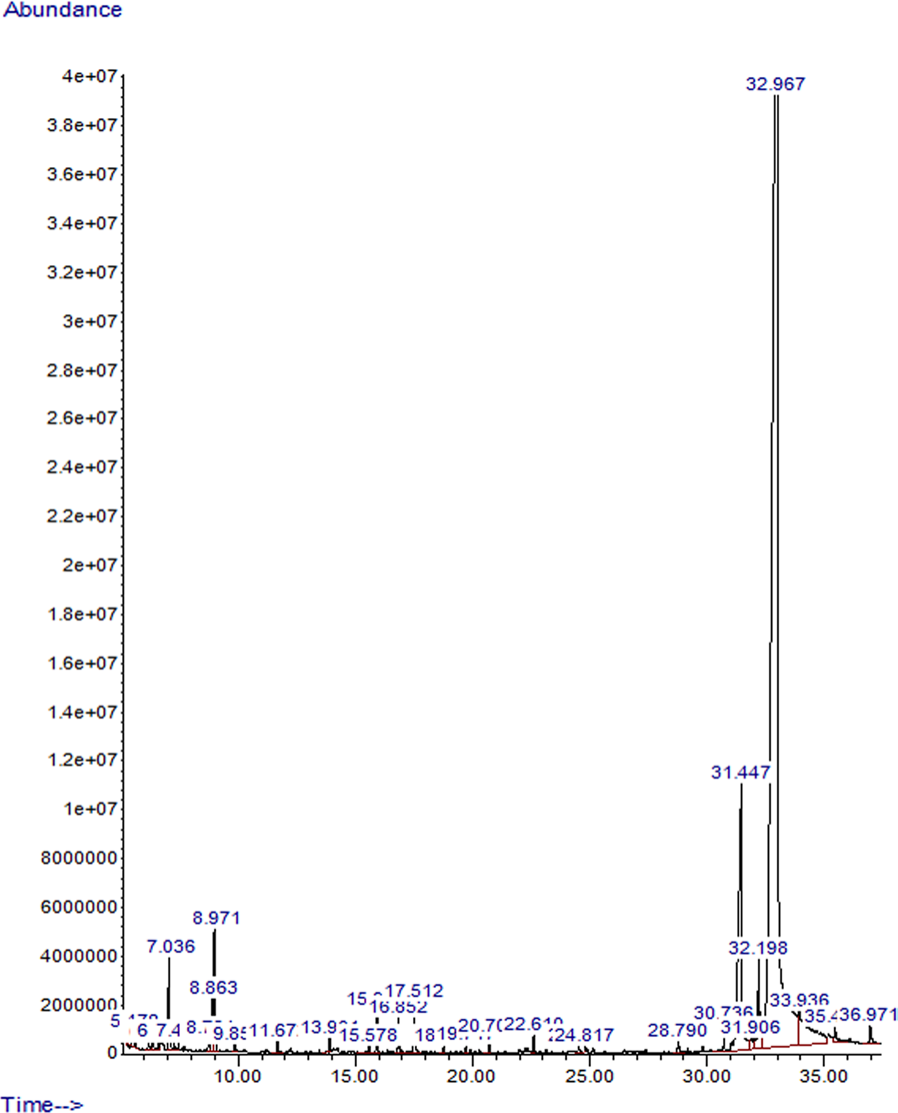
Chromatogram of phytochemicals in the hexane extract of *N. sativa* seed oil

The GC–MS analysis of *N. sativa* seed oil gave 27 peaks for the compounds detected, and the chromatogram is shown in Fig. 2.

The elution information of the compounds and their therapeutic uses are summarized in Table 1.

**Table 1:** Phytochemical compounds identified in *N. sativa* seed oil

Peak	% wt	Name of compound	PubMed CID	Therapeutic uses
1	0.22	1,2-dimethylcyclopentan-1-ol	551296	Still unknown
2	0.14	α-thujene	17868	Anti-inflammatory; anti-arthritic; antimicrobial
3	0.19	α-pinene	6654	Anti-inflammatory; antioxidant; anticancer; antimicrobial
4	0.13	1,3-dimethylcyclohexanol	558957	Still unknown
5	1.30	(*Z*)-hept-2-enal	5362616	Still unknown
6	0.23	β-pinene	14896	Antimicrobial
7	0.17	1-cynaopyrrolidine	73737	Still unknown
8	0.63	m-cymene	10812	Anti-inflammatory
9	1.61	D-Limonene	440917	Anti-inflammatory; anticancer; antimicrobial
10	0.14	γ-Terpinene	7461	Anti-inflammatory; antioxidant; antimicrobial
11	0.18	Trans-4-methoxy thujane	–	Antimicrobial
12	0.15	Terpineol	17100	Antioxidant; anticancer; anticonvulsant; antiulcer; antihypertensive; anti-nociceptive
13	0.11	Thymoquinone	10281	Anti-inflammatory; hepatoprotective; antioxidant; anticancer; cytotoxic
14	0.52	2-decenal, (E)-	5283345	Antinematicidal
15	1.39	2,4-decadienal, (E,E)-	5283349	Antinematicidal
16	0.08	Geranyl-2-methylbutyrate	6437648	Still unknown
17	0.09	Tetradecane	12389	Antimicrobial
18	0.20	α-bergamotene	86608	Antimicrobial
19	0.20	β-bisabolene	10104370	Antimicrobial; Anti-ulcer
20	0.12	Caryophyllene oxide	1742210	Anti-inflammatory; antimicrobial; antiparasitic; analgesic
21	0.06	Hexadecane	11006	Antibacterial
22	0.22	Myristic acid	11005	Antimycomicrobial
23	0.25	Methyl palmitate	8181	Anti-inflammatory
24	10.00	Palmitic acid	985	Antioxidant; antibacterial; anti-inflammatory; antitumor
25	1.62	Methyl linoleate	5284421	Antioxidant
26	79.61	Linoleic acid	5280450	Antidiabetic
27	0.44	9,17-Octadecadienal, (Z)-	5365667	Anti-inflammatory; antioxidant

**Fig. 3:**
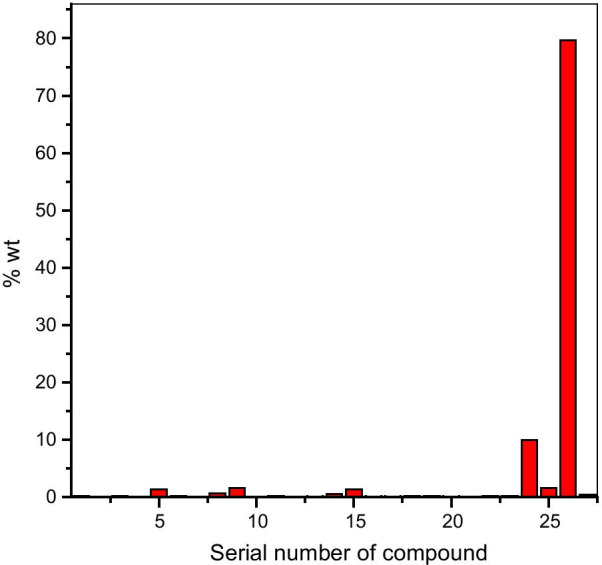
Abundance of components in N. sativa oil

The phytochemical components were present in the order linoleic acid > palmitic acid > methyl linoleate > D-limonene > 2,4-decadienal, (E,E)- > (*Z*)-hept-2-enal > m-cymene > 2-decenal, (E)- > 9,17-octadecadienal, (Z)- > methyl palmitate > β-pinene > 1,2-dimethylcyclopentan-1-ol = myristic acid > α-bergamotene = β-bisabolene > α-pinene > trans-4-methoxy thujane > 1-cynaopyrrolidine > terpineol > α-thujene = γ-terpinene > 1,3-dimethylcyclohexanol > caryophyllene oxide > thymoquinone > tetradecane > geranyl-2-methylbutyrate > hexadecane (Fig. 3). About 90% of the entire oil was comprised of linoleic acid (79.61%) and palmitic acid (10%).

The binding affinities of the compounds from the oil extract to the SARS-CoV-2 receptors are shown in Table 2.

**Table 2:**
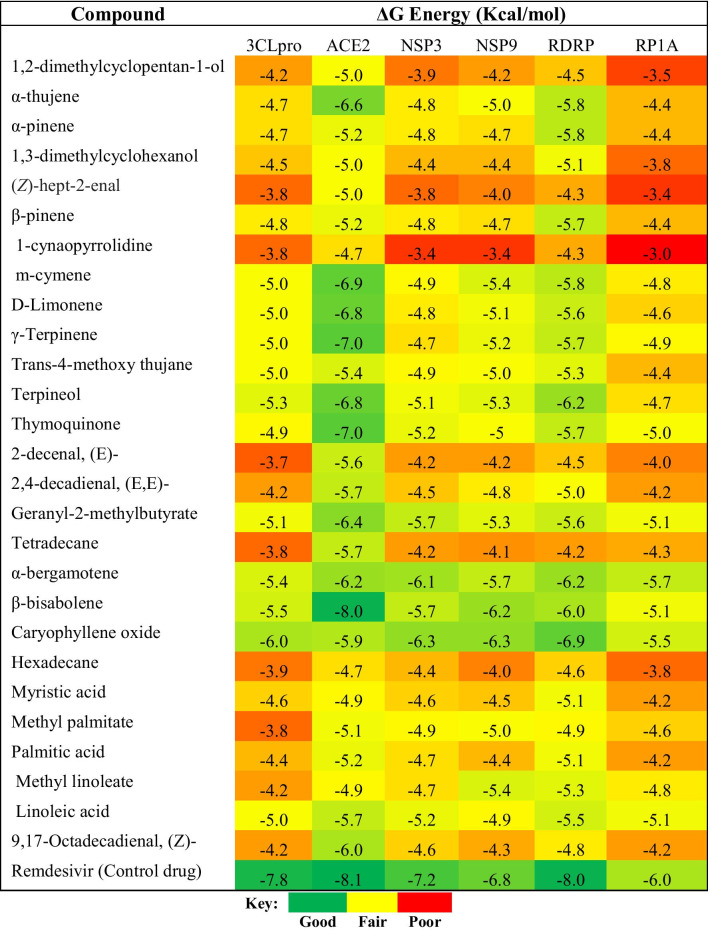
Binding affinities of compounds in the oil of *N. sativa* seed for SARS-CoV-2 proteins

## Discussion

Most of the identified phytochemicals in *N. sativa* seed oil have previously been reported to show antidiabetic, anti-inflammatory, antioxidant, anticancer, antimicrobial, anti-arthritic, anticonvulsant, antiulcer, antihypertensive, anti-nociceptive, hepatoprotective, cytotoxic, antimycomicrobial, antiparasitic, and analgesic activities (Marques et al. 2019; Caboni et al. 2012; Begum et al. 2016; Lang and Buchbauer 2012; Balamurugan et al. 2013; Muniyan and Gurunathan 2016; Saeed et al. 2012; Zhao et al. 2018; Adeoye-Isijola et al. 2018; Wu et al. 2017; Chavan et al. 2009; Yimer et al. 2019; Hadi et al. 2016; Engels and Brinkmann 2017). These compounds showed varying degrees of binding on the SARS-CoV-2 protein targets (Table 2), as revealed by the change in Gibb’s free energy values (ΔG) (Duru and Duru 2020). The FDA emergency approved drug Remdesivir (Williamson et al. 2020), administered on adults and children with suspected or confirmed cases of COVID-19, was used as a control to identify compounds with good binding affinities for the studied COVID-19 proteins. Caryophyllene oxide had the best binding affinities on 3CLpro (− 6.0 kcal/mol), NSP3 (− 6.3 kcal/mol), NSP9 (− 6.3 kcal/mol), and RDRP (− 6.9 kcal/mol) targets. The compound α-bergamotene gave the best binding affinity on RPIA (5.7 kcal/mol), while β-bisabolene displayed an excellent binding affinity on the ACE2 protein target. These values were very close to those obtained using the antiviral control drug. The binding affinity of β-bisabolene (− 8.0 kcal/mol) was almost similar to Remdesivir (− 8.1 kcal/mol) on the ACE2 target. This observation is an indication that β-bisabolene could prevent the interaction of the spike glycoprotein of the virus with the ACE2 protein of the human host cell, thereby preventing infection of the host.

**Fig. 4:**
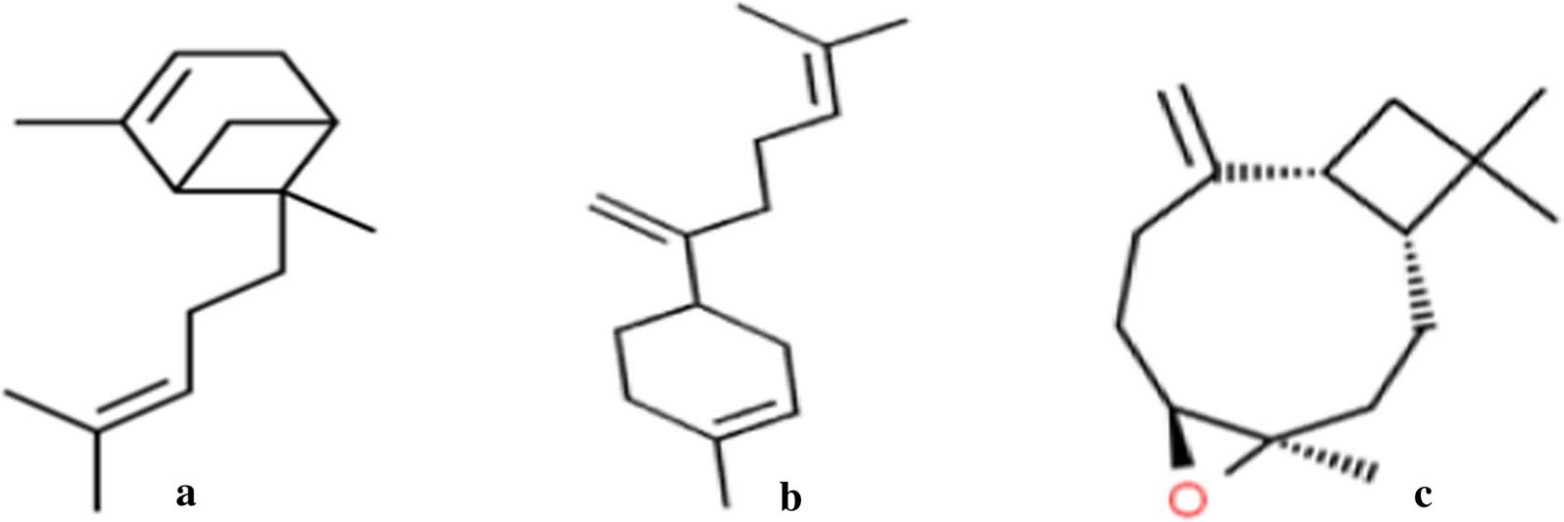
Structures of **a** α-bergamotene, **b** β-bisabolene, **c** Caryophyllene oxide

The structures of the compounds with the highest docking scores for the studied proteins are shown in Fig. 4. The absorption, distribution, metabolism, excretion, and toxicity (ADMET) properties which show their pharmacokinetics and pharmacodynamics properties are summarized in Table 3. Lipinski's rule of five, which is determined by a complex balance of various molecular properties and structural features such as lipophilicity, electronic distribution, hydrogen bonding characteristics, molecule size, and flexibility, as well as the presence of various pharmacophores that influence the behavior of a molecule in a living organism, were used to predict the drug likeliness of the compounds. A good drug candidate should not violate more than one of the rules (Lipinski 2016).

**Table 3:** ADMET properties of α-bergamotene, β-bisabolene, and Caryophyllene oxide relative to Remdesivir

ADMET properties	α-bergamotene	β-bisabolene	Caryophyllene oxide	Remdesivir
Molecular weight	204.36	204.36	220.36	602.56
WlogP	4.7	5.0	3.9	2.2
H-bond acceptor	0	0	1	13
H-bond donor	0	0	0	4
Rotatable bonds	3	3	0	13
Blood–brain barrier	+	+	+	+
Carcinogenicity (binary)	−	−	−	−
Human intestinal absorption	+	+	+	+
Acute oral toxicity	2.32	2.05	2.24	3.43
Water solubility	− 4.97	− 4.89	− 3.45	− 4.12

The molecular weights of the compounds were < 500, and their hydrophobicity (log P) did not exceed 5. The hydrogen bond donor ($\le$ 5 hydrogens) and hydrogen bond acceptor (not more than 10 hydrogens) of the compounds were in line with the rule. The rotatable bonds (not more than 3) were in line with the rule of three, and their acute oral toxicity values were well below 5 mg/kg. Caryophyllene oxide is soluble in water, while α-bergamotene and β-bisabolene are moderately soluble. The human intestinal adsorption and blood–brain barrier of the compounds were very high, and the compounds were not carcinogenic. These results are indications that α-bergamotene, β-bisabolene, and caryophyllene oxide are excellent drug candidates (Zhong 2017).

**Fig. 5:**
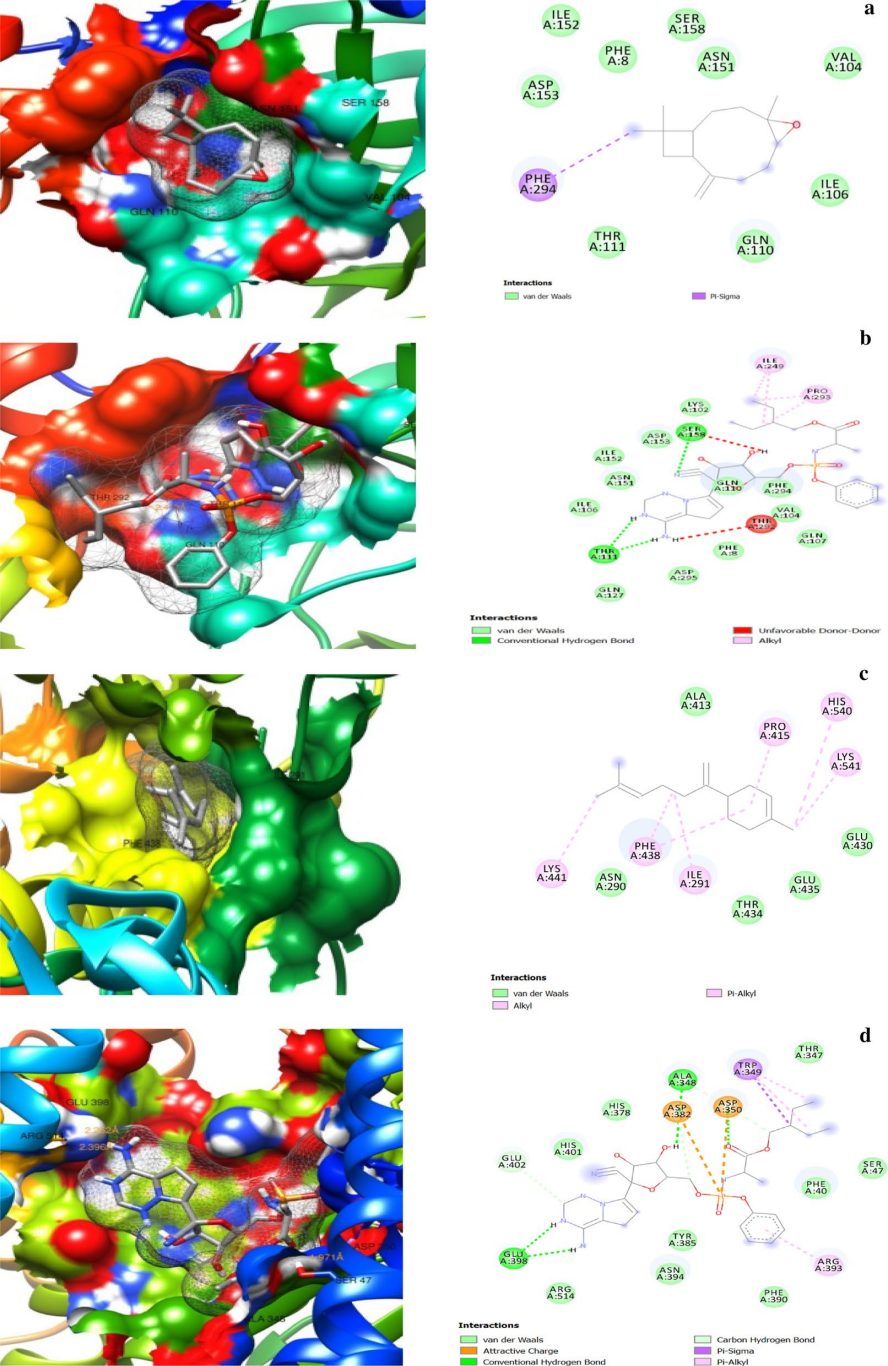

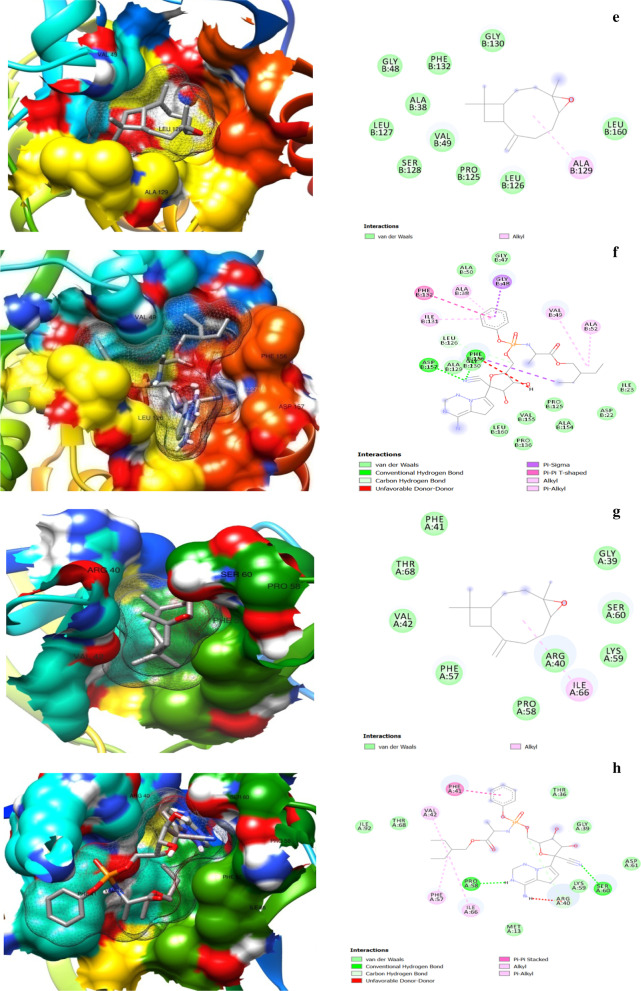

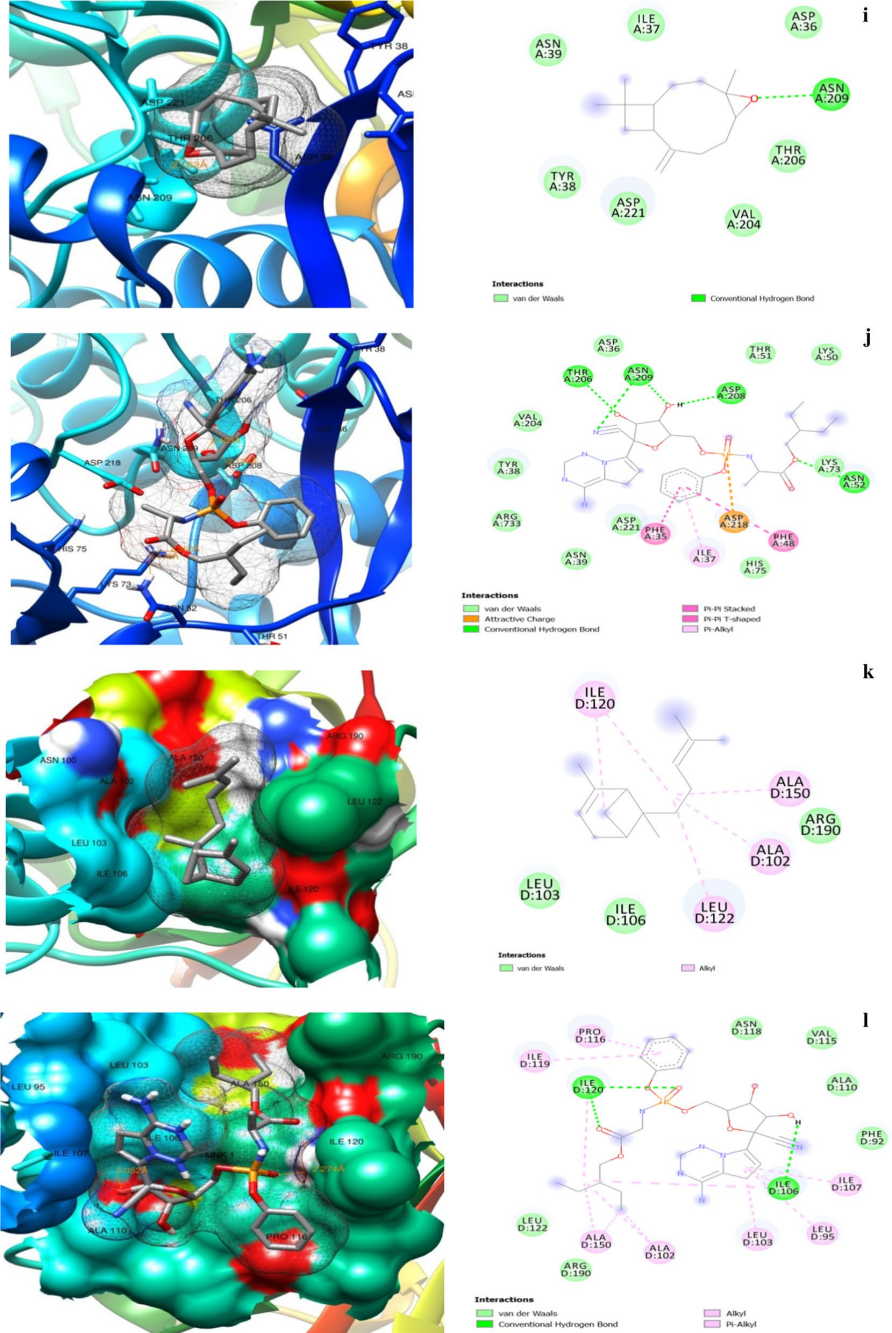
3D (left) and 2D (right) views of molecular interactions of **a** 3CLpro-caryophyllene oxide. **b** 3CLpro-Remdesivir. **c** ACE2-bisabolene. **d** ACE2-Remdesivir. **e** NSP3-caryophyllene oxide. **f** NSP3-Remdesivir. **g** NSP9-caryophyllene oxide. **h** NSP9-Remdesivir. **i** RDRP-cryophyllene oxide. **j** RDRP-Remdesivir. **k** RP1A-bergamotene. **l** RP1A-Remdesivir complexes

The 3D and 2D protein–ligand interaction images for α-bergamotene, β-bisabolene, and caryophyllene oxide are shown in Fig. 5. The amino acid residues involved in the interactions with the compounds and the control drug Remdesivir are depicted in Table 4.

**Table 4:** Protein residue interactions with the compounds and Remdesivir

Protein–ligand complex	Interacting residues						
	Hydrogen bond	Unfavorable donor–donor	Pi-sigma	Pi-alkyl	Alkyl	Carbon–hydrogen	Attractive charge
3CLpro-Caryopyellene oxide	–	–	PHE294	–	–	–	–
3CLpro-Remdesivir	SER158; THR111	SER158; THR292	–	–	1LE249; PRO293	–	–
ACE2- β-bisabolene	–	–	–	HIS450; LYS541; LYS441	HIS540; PRO415; PHE438; ILE291	–	–
ACE2-Remdesivir	GLU398; ALA348; ASP350	–	TRP349	TRP349; ARG393	–	GLU402; ALA348; ASP382	ASP382; ASP350
NSP3-Cryophyllene oxide	–	–	–	–	ALA129	–	–
NSP3-Remdesivir	ASP157; PHE156	PHE156	GLY48; PHE156	ALA38; ILE131	VAL49; ALA52	LEU126	–
NSP9-Cryophyllene oxide	–	–	–	–	ILE68	–	–
NSP9-Remdesivir	PRO58; SER60	ARG40	–	VAL42; PHE56	ILE66	ARG40	–
RDRP-Cryophyllene oxide	ASN209	–	–	–	–	–	–
RDRP-Remdesivir	ASN52; THR206; ASN209; ASP208	–	–	ILE37	–	–	ASP218
RP1A- α-bergamotene	˗	–	–	–	ILE120; ALA150; ALA102; LEU122	–	–
RP1A-Remdesivir	ILE120; ILE106	–	–	PRO116; ILE119; ILE107; LEU95; LEU103	ILE120; ILE106; ALA150; ALA102	–	–

The hydrogen bond, pi-sigma, pi-alkyl, and alkyl type interactions were the main forces that held the compounds in the protein pockets. At least two hydrogen bonds were observed in all the protein–Remdesivir interactions, and one was found only in the RDRP-caryophyllene oxide interaction. The protein–Remdesivir interactions in all the protein pockets had at least two hydrogen bonds and the other interacting forces, in the pockets of the studied proteins. Caryophyllene oxide and Remdesivir interacted with ASN 209 by hydrogen bonding in RDRP, while α-bergamotene and Remdesivir interacted by alkyl forces with ILE 120, ALA 150, and ALA 102. These suggested that the mechanism of inhibition of the proteins at these sites by caryophyllene oxide and α-bergamotene was similar to the action of Remdesivir at those sites.

## Conclusions

The phytochemical composition and binding affinity of compounds in the oil extract from *Nigella sativa* seed on SARS-CoV-2 molecular targets were investigated. The GC–MS analysis of the oil showed that it contained many compounds with known biological and therapeutic activities. The docking studies of these compounds against some major targets on the SARS-CoV-2 revealed that caryophyllene oxide, α-bergamotene, and β-bisabolene were promising candidates in the inhibition of the activity of these proteins. Their ADMET results further indicated that they would have good pharmacological and pharmacodynamic properties in the human system. There is therefore need for further in vitro, in vivo, and clinical trials to validate the potentials of these compounds in the eradication of the coronavirus pandemic.

## Data Availability

All data generated or analyzed during this study are included in this published article.

## Abbreviations

COVID-19: Coronavirus disease 2019
SARS-CoV-2: Severe acute respiratory syndrome coronavirus 2
GC–MS: Gas chromatography-mass spectrometer
MSD: Mass selective detector
NIST: National Institute of Standards and Technology
RP1A: Replicase polyprotein
NSP9: Nonstructural protein 9
NSP3: Nonstructural protein 3
3CLpro: 3-Chymotrypsin-like protease
RDRP: RNA-dependent RNA polymerase
ACE2: Angiotensin-converting enzyme
CASTp: Computed atlas for surface topography of proteins
ADMET: Absorption, distribution, metabolism and elimination and toxicity
FDA: Food and drug administration
TYR: Tyrosine
MET: Methionine
TRP: Tryptophan
ILE: Isoleusine
SER: Serine
ALA: Alanine
PHE: Phenylalanine
THR: Threonine
VAL: Valine
ARG: Arginine
PRO: Proline
LEU: Leucine
HIS: Histidine
ASN: Asparagine
GLY: Glycine
LYS: Lysine
GLU: Glutathione

## References

[CR1] Abdelmeguid NE, Fakhoury R, Kamal SM, Al Wafai RJ (2010). Effects of *Nigella sativa* and thymoquinone on biochemical and subcellular changes in pancreatic β-cells of streptozotocin-induced diabetic rats. J Diabetes.

[CR2] Adeoye-Isijola MO, Olajuyigbe OO, Jonathan SG, Coopoosamy RM (2018). Bioactive compounds in ethanol extract of *Lentinus squarrosulus* mont - a Nigerian medicinal macrofungus. Afr J Tradit Complement Altern Med.

[CR3] Ahmad A, Husain A, Mujeeb M, Khan SA, Najmi AK, Siddique NA (2013). A review on therapeutic potential of Nigella sativa: A miracle herb. Asian Pac J Trop Biomed.

[CR4] Al YM (1986). Phytochemical studies of the plants used in traditional medicine of Saudi Arabia. Fitoterapia.

[CR5] Awadalla EA (2012). Ameliorative effect of the crude oil of the *Nigella sativa* on oxidative stress induced in rat testes by cisplatin treatment. Biomed Prev Nutr.

[CR6] Balamurugan M, Selvam GG, Thinakaran T, Sivakumar K. Biochemical study and GC-MS analysis of Hypnea musciformis (Wulf.) Lamouroux Am-Euras. J Sci Res. 2013; 8(3):117–123.

[CR7] Begum IF, Mohankumar R, Jeevan M, Ramani K (2016). GC-MS analysis of bioactive molecules derived from *Paracoccus pantotrophus* FMR19 and the antimicrobial activity against bacterial pathogens and MDROs. Indian J Microbiol.

[CR8] BIOVIA, Dassault Systemes, San Diego, Discovery studio modeling environment, 2020.

[CR9] Caboni P, Ntalli NG, Aissani N, Cavoski I, Angioni A (2012). Nematicidal activity of (E, E)-2,4-decadienal and (E)-2-decenal from* Ailanthus altissim*a against* Meloidogyne javanic*a. J Agric Food Chem.

[CR10] Chavan M, Wakte P, Shinde D (2010). Analgesic and anti-inflammatory activity of cryophyllene oxide from *Annona squamosa* leaf bark. Phytomedicine.

[CR11] Datta AK, Saha A, Bhattacharya A, Mandal A, Paul R Sonali SS. Black cumin (Nigella sativa L.) - A review. J Plant Dev Sci. 2012; 4(1):1–43.

[CR12] Duru CE (2020). Mineral and phytochemical evaluation of *Zea mays* husk. Sci Afr.

[CR13] Duru CE, Duru IA, Bilar A (2020). Computational investigation of sugar fermentation inhibition by bergenin at the pyruvate decarboxylate isoenzyme 1 target of *Scharomyces cervisiae*. J Med Plants Stud.

[CR14] Duru IA, Duru CE (2020). Molecular docking of compounds in the essential oil of *Ocimium gratissimum* leaf against PIM-1 kinase of *Escherichia coli*. Ej-Chem.

[CR15] El-Sawi SA, Maamoun AA, Salama AH, Farghaly AA (2020). Chemical profiling of *Thevetia peruviana* leaves cytotoxic active extracts enhanced by microemulsion formulation. Bull Natl Res Cent.

[CR16] Engel G, Brinkmann J (2017). Nigella- *Nigella sativa*, Ranunculaceae. HerbalGram.

[CR17] Gao Y, Yan L, Huang Y, Liu F, Zhao Y, Cao L (2020). Structure of the RNA-dependent RNA polymerase from COVID-19 virus. Science.

[CR18] Guy JL, Jackson RM, Jensen HA, Hooper NM, Turner AJ (2005). Identification of critical active-site residues in angiotensin-converting enzyme-2 (ACE2) by site-directed mutagenesis. FEBS J.

[CR19] Hadi MY, Mohammed GJ, Hameed IH (2016). Analysis of bioactive chemical compounds of *Nigella sativa* using gas chromatography-mass spectrometry. J Pharmacognosy Phytother.

[CR20] http://sts.bioe.uic.edu/castp/index.html?j_5f45dd381f58d

[CR21] Jin Z, Du X, Xu Y, Deng Y, Liu M, Zhao Y (2020). Structure of M^pro^ from SARS-CoV-2 and discovery of its inhibitors. Nature.

[CR22] Johnson TO, Odoh KD, Akinanmi AO, Adegboyega AE (2020). Biochemical evaluation and molecular docking assessment of the anti-inflammatory potential of *Phyllanthus nivosus* leaf against ulcerative colitis. Heliyon.

[CR23] Kamil ZH (2013). Spectacular black seeds (Nigella sativa): Medical importance review. Med J Babylon.

[CR24] Konkolova E, Klima M, Nencka R, Boura E (2020). Structural analysis of the putative SARS-CoV-2 primase complex. J Struct Biol.

[CR25] Kumar Y, Singh H, Patel CN (2020). In silico prediction of potential inhibitors for the Main protease of SARS-CoV-2 using molecular docking and dynamics simulation based drug-repurposing. J Infect Public Health. 2020; 13(9):1210–1223. 10.1016/j.jiph.2020.06.016 PMC7297718 32561274

[CR26] Lang G, Buchbauer G (2012). A review on recent research results (2008–2010) on essential oils as antimicrobials and antifungals. A review Flavour Fragr J.

[CR27] Lipinski CA (2016). Rule of five in 2015 and beyond: Target and ligand structural limitations, ligand chemistry structure and drug discovery project decisions. Adv Drug Deliv Rev.

[CR28] Marques FM, Figueira MM, Schmitt EFP, Kondratyuk TP, Endringer DC, Scherer R (2019). In vitro anti-inflammatory activity of terpenes via suppression of superoxide and nitric oxide generation and the NF-κB signalling pathway. Inflammopharmacology.

[CR29] Michalska K, Kim Y, Jedrzejczak R, Maltseva NI, Stols L, Endres M, Joachimiak A (2020). Crystal structures of SARS-CoV-2 ADP-ribose phosphatase: from the apo form to ligand complexes. IUCrJ.

[CR30] Muniyan R, Gurunathan J (2016). Lauric acid and myristic acid from *Allium sativum* inhibit the growth of Mycobacterium tuberculosis H37Ra: in silico analysis reveals possible binding to protein kinase B. Pharm Biol.

[CR31] Naz H (2011). Nigella sativa: the miraculous herb. Pak J Biochem Mol Biol.

[CR32] Peng L, Liu A, Shen Y, Xu H, Yang S, Ying X (2013). Antitumor and anti-angiogenesis effects of thymoquinone on osteosarcoma through the NF-κB pathway. Oncol Rep.

[CR33] Pettersen EF, Goddard TD, Huang CC, Couch GS, Greenblatt DM, Meng EC, Ferrin TE (2004). UCSF Chimera- a visualization system for exploratory research and analysis. J Comput Chem.

[CR34] Punch Newspaper, April 7, 2020. https://healthwise.punchng.com/i-fought-covid-19-with-vitamin-c-black-seed-oil-mixed-with-honey-gov-makinde-2/

[CR35] Saeed NM, El-Demerdash E, Abdel-Rahman HM, Algandaby MM, Al-Abbasi FA, Abdel-Naim AB. Anti-inflammatory activity of methyl palmitate and ethyl palmitate in different experimental rat models. Toxicol Appl Pharmacol. 2012; 1:264(1):84–93. 10.1016/j.taap.2012.07.020 22842335

[CR36] Shariq IM, Israil AM, Iqbal A, Brijesh P. Morpho-physiological characterization of seeds and seedlings of *Nigella sativa* Linn.: Study on Indian germplasm. Int Res J Biol Sci. 2015; 4(4):38–42.

[CR37] Sharma N, Ahirwar D, Jhade D, Gupta S (2009). Medicinal and phamacological potential of *Nigella sativa*: A review. Ethnobot Rev.

[CR38] Sultan MT, Butt MS, Karim R, Iqbal SZ, Ahmad S, Zia-UI-Haq M (2014). Effect of Nigella sativa fixed and essential oils on antioxidant status, hepatic enzymes, and immunity in streptozotocin induced diabetes mellitus. BMC Complement Altern Med.

[CR39] Tan K, Kim Y, Jedrzejczak R, Maltseva N, Endres M, Michalska K, Joachimiak A. The crystal structure of Nsp9 RNA binding protein of SARS CoV-2. Center for Structural Genomics of Infectious Diseases (CSGID); 2020. https://doi.org/10.2210/pdb6W4B/pdb

[CR40] Tian W, Chen C, Lei X, Zhao J, Liang J. CASTp 3.0: computed atlas of surface topography of proteins. Nucleic Acids Res. 2018; 46:363–367. 10.1093/nar/gky473 PMC6031066 29860391

[CR41] Tsao YC, Chang YJ, Wang CH, Chen L (2020). Discovery of isoplumbagin as a novel NQO1 substrate and anti-cancer quinone. Int J Mol Sci.

[CR42] Umar S, Zargan J, Umar K, Ahmad S, Katiyar CK, Khan HA (2012). Modulation of the oxidative stress and inflammatory cytokine response by thymoquinone in the collagen induced arthritis in Wistar rats. Chem Biol Interact.

[CR43] Vavilov NI, Dorofeev VF (1992). Origin and Geography of Cultivated Plants.

[CR44] Wang Q, Zhang Y, Wu L, Niu S, Song C, Zhang Z (2020). Structural and functional basis of SARS-CoV-2 entry by using human ACE2. Cell.

[CR45] WHO. Novel coronavirus (2019-nCoV), 2020. https://www.euro.who.int/en/health-topics/health-emergencies/novel-coronavirus-2019-ncov_old.

[CR46] Williamson BN, Feldmann F, Schwarz B, Meade-White K, Porter DP, Schulz J (2020). Clinical benefit of remdesivir in rhesus macaques infected with SARS-CoV-2. Nature.

[CR47] Wu JHY, Marklund M, Imamura F, Tintle N, Ardisson Korat AV, de Goede J (2017). Omega-6 fatty acid biomarkers and incident type 2 diabetes: pooled analysis of individual-level data for 39 740 adults from 20 prospective cohort studies. Lancet Diabetes Endocrinol.

[CR48] Yang H, Lou C, Sun L, Li J, Cai Y, Wang Z, et al. admetSAR 2.0: Wed-service for prediction and optimization of chemical ADMET properties. Bioinformatics (Oxford, England), 2019; 35(6):1067–1069. 10.1093/bioinformatics/bty707 30165565

[CR49] Yimer EM, Tuem KB, Karim A, Ur-Rehman N, Anwar F. *Nigella sativa* L. (Black Cumin): A promising natural remedy for wide range of illnesses. Evid Based Complement Alternat Med. 2019; 1528635. 10.1155/2019/1528635 10.1155/2019/1528635 PMC6535880 31214267

[CR50] Zhao Z, Vavrusova M, Skibsted LH (2018). Antioxidant activity and calcium binding of isomeric hydroxybenzoates. J Food Drug Anal.

[CR51] Zhong HA, Grover A (2017). ADMET properties: overview and current topics. Drug design: principles and applications.

